# Intranuclear Peripheral Overexpression of Pituitary-Tumor-Transforming Gene 1: Immunohistochemical Biomarker of Lymph Node Involvement in Testicular Seminoma

**DOI:** 10.3390/cancers18071163

**Published:** 2026-04-04

**Authors:** Edoardo Vergani, Francesco Pierconti, Carlotta Pozza, Elisabetta Merenda, Paola Girardi, Marta Tenuta, Roberta Benvenuto, Emanuela Teveroni, Gaetano Gulino, Giorgio Franco, Fabio Massimo Magliocca, Bernardo Rocco, Andrea Isidori, Alfredo Pontecorvi, Domenico Milardi

**Affiliations:** 1 Unit of Internal Medicine, Endocrinology and Diabetology, Fondazione Policlinico Universitario Agostino Gemelli IRCCS, Università Cattolica del Sacro Cuore, 00168 Rome, Italy; paola.girardi@guest.policlinicogemelli.it (P.G.); alfredo.pontecorvi@unicatt.it (A.P.); domenico.milardi@policlinicogemelli.it (D.M.); 2 International Scientific Institute Paolo VI, 00168 Rome, Italy; 3 Division of Pathology and Histology, School of Medicine, Università Cattolica del Sacro Cuore, 00168 Rome, Italy; francesco.pierconti@policlinicogemelli.it (F.P.); elisabetta.merenda@guest.policlinicogemelli.it (E.M.); roberta.benvenuto@policlinicogemelli.it (R.B.); 4 Department of Experimental Medicine, Sapienza University of Rome, Viale Regina Elena 324, 00161 Rome, Italy; carlotta.pozza@uniroma1.it (C.P.); marta.tenuta@gmail.com (M.T.); andrea.isidori@uniroma1.it (A.I.); 5 Department of Laboratory and Infectious Diseases, Fondazione Policlinico Universitario Agostino Gemelli IRCCS, 00168 Rome, Italy; emanuela.teveroni@guest.policlinicogemelli.it; 6 Urology Department, Fondazione Policlinico Universitario Agostino Gemelli IRCCS, 00168 Rome, Italy; gaetano.gulino@policlinicogemelli.it (G.G.); bernardo.rocco@policlinicogemelli.it (B.R.); 7 Department of Maternal and Child Health and Urological Sciences, Sapienza University, 00185 Rome, Italy; giorgio.franco@uniroma1.it; 8 Department of Radiological, Oncological and Pathological Sciences, Sapienza University of Rome, 00185 Rome, Italy; fabiomassimo.magliocca@uniroma1.it

**Keywords:** testicular seminoma, lymph node metastasis, PTTG1, biomarker, prognostic stratification, personalized medicine

## Abstract

**Background/Objectives**: Testicular germ cell tumors, particularly seminoma, represent the leading cause of cancer in men aged 15–40 years. The decision about adjuvant therapy relies on histological features with uncertain prognostic value. The Pituitary-Tumor-Transforming Gene 1 (PTTG1), which encodes the securin protein, is crucial in sister chromatid separation. Our previous in vitro studies demonstrated that PTTG1 nuclear expression promotes invasiveness, dedifferentiation, and neolymphangiogenesis in testicular seminoma. **Methods**: We conducted a hypothesis-generating retrospective observational study on 51 patients (aged 23–68) with testicular seminoma, with (43%) or without (57%) lymph node involvement, evaluating potential correlations between PTTG1 and currently known prognostic factors. Clinical and pathological data were collected, including lymph node involvement, recurrence, necrosis, rete testis invasion, vascular invasion, and adipose tissue invasion. An immunohistochemical scoring system assessing intranuclear PTTG1 expression was developed. **Results**: The PTTG1 score was related to lymph node metastasis and adipose tissue invasion. ROC curve analysis showed that the PTTG1 immunohistochemical score showed good discriminatory ability in identifying lymph node involvement (AUC = 0.939); the optimal cut-off was 4.0 (sensitivity 90.5%; specificity 57.1%), while the ROC curve for adipose tissue invasion was inadequate. Lymph node metastasis also correlated with necrosis; however, logistic regression confirmed that PTTG1 score was independently associated with nodal involvement (*p* = 0.002), regardless of tumor size and necrosis. **Conclusions**: Our findings suggest a correlation between PTTG1 expression and lymphadenopathy at diagnosis, independent of tumor size and T stage. It may reflect biological features associated with lymphatic dissemination and requires further investigation in larger prospective studies.

## Simple Summary

Testicular seminoma is the most common cancer in young adult men and generally has an excellent prognosis. However, a subset of patients develops lymph node metastases and may require more intensive treatment. Clinicians rely on histological features to estimate the risk of disease spread, but these factors are not always reliable. Pituitary-Tumor-Transforming Gene 1 (PTTG1) is a protein involved in cell division and has been linked to tumor aggressiveness. In this study, we evaluated whether the expression of PTTG1 in tumor tissue could be associated with lymph node involvement in seminoma. We analyzed tumor samples from 51 patients using immunohistochemistry and developed a scoring system based on intranuclear PTTG1 expression. Higher PTTG1 scores were associated with the presence of lymph node metastases at diagnosis, independently of tumor size. These results suggest that PTTG1 immunohistochemical is associated with lymph node metastasis but this finding requires prospective validation in larger cohorts.

## 1. Introduction

Testicular germ cell tumors (TGCTs), particularly seminoma, represent the leading cause of cancer in men aged 15–40 years [1]. Scrotal ultrasound guides the initial assessment of testicular lesions [2,3], along with serum tumor markers (alpha-fetoprotein—AFP, beta-human chorionic gonadotropin—β-hCG, lactate dehydrogenase—LDH), which remain essential for diagnosis, staging, and follow-up. In clinical practice, features such as tumor size or histological findings (necrosis, rete testis invasion, angioinvasion, adipose tissue invasion, and the presence of funicular involvement) are used to identify stage I seminomas with a higher risk of recurrence [4,5]. The overall prognosis is excellent; however, it worsens in presence of lymph node involvement or distant metastases. The presence of metastatic lymphadenopathy upgrades the disease from stage I to stage II, requiring a shift in treatment strategy. In such cases, more aggressive therapeutic options (multi-cycle cisPlatin–Etoposide–Bleomycin PEB chemotherapy, radiotherapy, or retroperitoneal lymphadenectomy) are warranted due to the higher recurrence rate and less favorable prognoses associated with this stage [6,7]. A thorough understanding of TGCTs biology would be essential to tailor the medical treatment; however, it remains far from complete [8]. In recent decades, literature focused on genetic alterations, such as those regarding KIT, KRAS and NRAS, or the gain of chromosome 12 p material, epigenetic changes (DNA hypomethylation in seminomas, and altered expression of micro RNAs) and the expression of the so-called cancer–testis antigens, which can serve as future potential biomarkers [9,10,11,12].

In this context, the proto-oncogene Pituitary-Tumor-Transforming Gene 1 (PTTG1) emerges as a possible relevant player in testicular seminomas [13]. High intranuclear levels of this oncogene at the tumor periphery [14], a more invasive-prone area, have been associated with mechanisms of invasiveness (increased expression of matrix metalloproteinase and dedifferentiation programs (epithelial–mesenchymal transition) in neoplastic germ cells in vitro [15,16]. These mechanisms could influence the biological behavior of the tumor and, when PTTG1 is overexpressed and not constrained to the cytoplasm by spectrin, might explain a greater degree of aggressiveness [17,18]. While molecular biology techniques from our previous studies offer an optimal means of investigating PTTG1-related pathways, their routine clinical application is limited by time requirements, the need for specialized laboratories, and trained personnel. Conversely, histopathological analysis remains a well-established and essential step in tumor characterization. For now, integrating PTTG1 assessment into clinical practice would necessarily rely on immunohistochemical analysis of paraffin-embedded human tissue. The aim of this study was therefore to explore, in a retrospective, hypothesis-generating setting, the relationship between intranuclear PTTG1 expression and the clinicopathological features of testicular seminoma, with particular focus on lymph node involvement. Given the hypothesized role of PTTG1 in tumor invasiveness and lymphatic dissemination, the study was designed to investigate whether PTTG1 expression reflects a biological phenotype associated with nodal spread.

## 2. Materials and Methods

A preliminary hypothesis-generating retrospective observational study was conducted following the principles of the Helsinki Declaration (2013 revision). The study protocol was reviewed and approved by the Institutional Review Board of the Territorial Ethics Committee of “Lazio area 3” (21 June 2024, protocol number: 4824). Patients were retrospectively identified from institutional databases and included based on the availability of adequate histological material and complete clinicopathological data. One of the primary aims of the study was to investigate the relationship between PTTG1 expression and lymph node involvement; therefore, the cohort included patients both with and without radiological evidence of nodal disease, resulting in a higher proportion of node-positive cases than typically observed in unselected seminoma populations. Fifty-one male patients, aged between 23 and 68 years, with histologically confirmed testicular seminoma, with or without lymph node involvement, were enrolled after providing written informed consent. All had undergone orchifunicolectomy at the Fondazione Policlinico Universitario Agostino Gemelli—IRCCS (Rome, Italy), or at the Policlinico Umberto I (Rome, Italy) between 2015 and 2024.

Before surgery, patients underwent diagnostic procedures to ensure proper staging of the neoplastic disease according to the TNM classification system [19], including clinical examination, testicular ultrasound, tumor marker assessment and CT scan of thorax and abdomen or MRI. The diagnosis of seminoma was established based on histopathological evaluation, supported by immunohistochemical analysis when required. In these cases, the tumors showed the expected immunophenotype, including PLAP and CD117 positivity. The cut-off values for serum markers used at our institution were as follows: β-hCG 0–5 mU/mL and AFP 0–9 ng/mL. Serum tumor markers were consistent with a diagnosis of seminoma and did not indicate non-seminomatous components.

Abdominal CT and MRI were both employed for the evaluation of lymphadenopathy. Lymph nodes were deemed positive if the short-axis diameter exceeded 1 cm and were in regions typical of seminoma spread (retroperitoneal, para-aortic, or interaortocaval). For less typical anatomical sites, such as obturator, inguino-femoral, and crural lymph nodes, lymphadenopathy was considered metastatic when radiological features were consistent with malignant involvement, including short-axis diameter above the established cut-off and/or additional suspicious characteristics such as central necrosis or loss of normal nodal architecture [6,7,20].

In the Supplementary Materials, Table S1 shows the localization of the metastatic lymph nodes (N+) for all the patients in our cohort.

The exclusion criteria included the presence of non-germ cell testicular lesions or non-seminomatous TGCTs, bilateral seminoma, testicular tumors smaller than 5 mm (a size potentially compromising accurate and complete histological diagnosis), and the presence of other active malignant neoplasms. To exclude the presence of non-seminomatous germ cell components, all tumors were extensively sampled for histological examination.

Recurrence was defined as the appearance of new disease after primary treatment during follow-up and did not include lymph node involvement present at diagnosis.

### 2.1. Immunohistochemistry

The archival cases were provided by the Unit of Pathological Anatomy at the Fondazione Policlinico Universitario Agostino Gemelli—IRCCS, Rome and by the Department of Radiological, Oncological and Pathological Sciences, Sapienza University of Rome, Italy.

The investigation was performed on testicular tissue fixed in formalin and embedded in paraffin. PTTG1 may play a pivotal role in testicular spermatogenesis, particularly in the differentiation and survival of germ cells [14]. Therefore, a sample of healthy tissue located approximately 2 cm from the tumor was used as a control. When this was not available, particularly in cases of large seminomas, normal testicular tissue obtained from our institutional tissue collection was used as a positive control for immunohistochemical analysis [21].

Morphological studies were done using hematoxylin-eosin staining. In addition, immunohistochemistry for placental alkaline phosphatase (PLAP), CD117 (KIT), and vimentin was performed on the analyzed samples.

As the primary monoclonal anti-PTTG1 antibody, we used SPM210 (Santa Cruz Biotechnology, Santa Cruz, CA, USA), which is capable of recognizing the whole PTTG1 protein. Immunohistochemistry for PLAP was performed using a pre-diluted rabbit polyclonal anti-placental alkaline phosphatase antibody (Signet Pathology Systems, Inc., Dedham, MA, USA; 45 min incubation). Immunohistochemistry for vimentin was performed using a pre-diluted mouse monoclonal anti-human vimentin antibody (Dako Corporation, Carpinteria, CA, USA; 10 min incubation). CD117 was identified using a pre-diluted rabbit polyclonal anti-human CD117 antibody (Dako Corporation, Carpinteria, CA, USA; 10 min incubation). For the PTTG1 expression study, 5 μm thick sections were mounted on “superfrost” polarized slides, dried and left overnight at 37 °C for optimal adhesion. Sections were then deparaffinized in xylene and rehydrated through decreasing concentrations of alcohol to water. For antigen retrieval, sections were treated with 0.01 molar citrate buffer at pH 6 and then cooled for about 20 min. To reduce the aspecific background in the endogenous peroxidases, slides were treated for 5 min with 0.3% H_2_O_2_. Sections were incubated overnight at 4 °C for PTTG1 (1:100 dilution). After PBS washes, slides were incubated for 20 min with Envision DAKO secondary antibody, followed by PBS washes. Diaminobenzidine (DAB) was used as chromogen, with final rinsing in tap water. Slides were then counterstained with hematoxylin for 10 s, dehydrated through increasing alcohol concentrations and xylene, and then mounted. The percentage of PTTG1-positive cells was evaluated both in the central area and at the periphery of the tumor. Predominant cytoplasmic or nuclear immunoreactivity was also assessed. According to our previous study, in seminomas larger than 2.5 cm, PTTG1 expression was significantly higher in the peripheral region compared to the central area. In contrast, this differential distribution of PTTG1-positive cells between peripheral and central regions was not observed in smaller seminomas (<2.5 cm) [14]. The percentage of positive cells (percentage score) was manually assessed by counting the positive stained cells, including cytoplasmatic and nuclear staining, in 10 High-Power Field (HPF, ×40). In seminomas with a diameter ≤ 2.5 cm, evaluation was performed in the peripheral tumor area only, whereas in tumors > 2.5 cm, both central and peripheral regions were analyzed. The mean value across the examined fields was calculated.

PTTG1 immunoreactivity was categorized into five levels based on the percentage of positive cells: 0: <10%, 1: 10–25%, 2: 25–50%, 3: 50–75%, 4: >75%.

**Figure 1:**
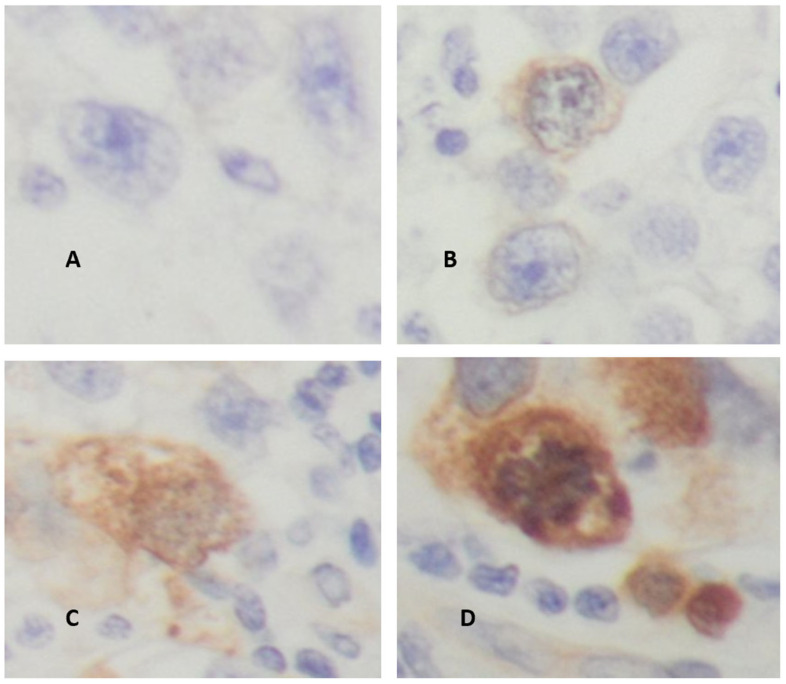
Immunohistochemistry staining of testicular seminoma tissues. (**A**) Negative staining for PTTG1 (40×). (**B**) Weak staining for PTTG1 (40×) (**C**) Moderate staining for PTTG1 (40×). (**D**) Strong staining for PTTG1 (40×).

The intensity score was graded on four levels according to staining: 0: no staining, 1: weak staining, 2: moderate staining, 3: strong staining. Figure 1 shows staining examples from seminoma tissue.

The PTTG1 immunohistochemical score was defined as:

overall score = percentage score × intensity score

Based on this score, samples were classified as negative (≤3), weakly positive (>3 to ≤6), or strongly positive (>6) [22,23]. All sections were independently evaluated by two investigators, blinded to patient outcomes. The final score reported in this study represents the mean of the two assessments.

**Figure 2:**
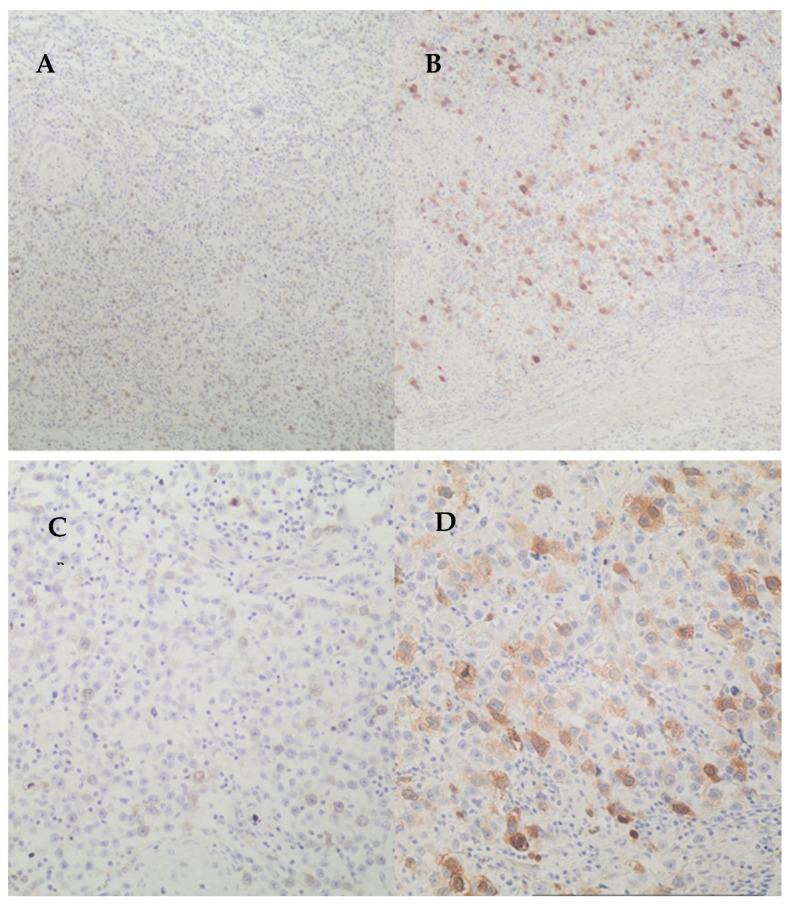
Immunohistochemistry staining of testicular seminoma tissues. (**A**) Testicular seminoma (weakly positive patient, score of 4), magnification 4×. (**B**) Testicular seminoma (strongly positive patient, score of 12), magnification 4×. (**C**) Testicular seminoma (weakly positive patient, score of 4), magnification 10×. (**D**) Testicular seminoma (strongly positive, score of 12), magnification 10×.

Figure 2 shows some examples of the immunohistochemistry staining obtained in the studied cohort.

### 2.2. Statistical Analysis

The statistical analysis was performed with Prism Graphpad 10.2.3 and Jasp 0.95.

All variables under study were summarized using descriptive statistical techniques. The D’Agostino-Pearson test was used to verify the normality of quantitative data distribution.

All the quantitative variables (PTTG1 score, age, BMI, tumor dimension) were normally distributed and were described as mean and standard deviation (SD). Qualitative variables were dichotomic (yes or no) and described as a percentage. The Pearson’s correlation test and the point-biserial were adopted to test correlations between variables. The student’s *t*-test was applied to verify differences between groups. A *p*-value less than 0.05 was considered statistically significant.

## 3. Results

Fifty-one male patients (mean age 40.6 ± 9.7 years) with histologically confirmed testicular seminoma, with or without lymph node involvement, were enrolled in the study. Table 1 shows the mean ± SD of the general features of our population.

As regards the PTTG1 immunohistochemical score, the median value was 4 (range 0–12), while the interquartile range was 2–7. Figure 2 shows via waterfall plot the PTTG1 immunohistochemical scores calculated in our population.

Table 2 describes the histopathological and clinical features of the 51 patients affected by testicular seminoma who were included in the study. Seminomas were predominantly pT2 (78%); metastatic lymphadenopathy was present in 43%; rete testis and vascular invasion were common (72% and 61%, respectively); necrosis and adipose/epididymal invasion occurred in 41% and recurrence was observed in 14% of evaluable patients (44/51). The most frequent treatments were single-shot carboplatin (31%), PEB (24%), and active surveillance (22%), with radiotherapy (10%) and surgery (2%) used less frequently.

The table reports tumor staging according to the TNM classification, metastatic lymphadenopathy, stage according to UICC 2016, key histopathological characteristics (including rete testis invasion, vascular invasion, necrosis, adipose tissue invasion, and spermatic cord involvement), treatment strategies applied, and recurrence rates observed during follow-up. Data are presented as absolute numbers and percentages.

The PTTG1 score correlated with lymph node involvement (*p* < 0.008, R^2^ 0.22, positive correlation) and adipose tissue involvement (*p* < 0.02, R^2^ 0.12, negative correlation). No significant association was observed between PTTG1 score and recurrence in our cohort.

**Figure 3:**
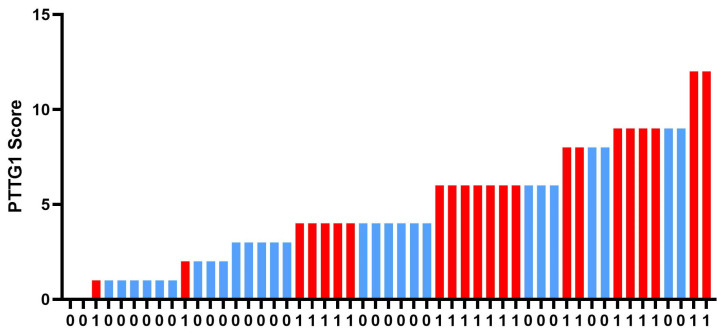
PTTG1 immunohistochemical score in the study population. This figure describes PTTG1 score in all patients. Red color was used for N+ patients, while blue color was used for N− patients.

Figure 3 shows the significantly different value of the PTTG1 score in N+ patients vs. N− ones, that was increased in patients with lymph node metastases, and patients with adipose tissue involvement vs. patients with no histological involvement of adipose tissue, that was decreased in the first group.

To evaluate the performance of the PTTG1 immunohistochemical score in identifying lymph node involvement, we performed a ROC curve analysis. The Youden index identified an optimal cut-off of 4.0 (AUC 0.939, 95%CI 0.889–0.988, *p* < 0.001), yielding high sensitivity (90.5%) with moderate specificity (57.1%), indicating useful discrimination for this endpoint. In contrast, for adipose tissue invasion, despite its positive correlation with PTTG1 immunohistochemical score, ROC curve analysis demonstrated a poor discriminative ability (AUC = 0.30 CI 0.16–0.45).

**Figure 4:**
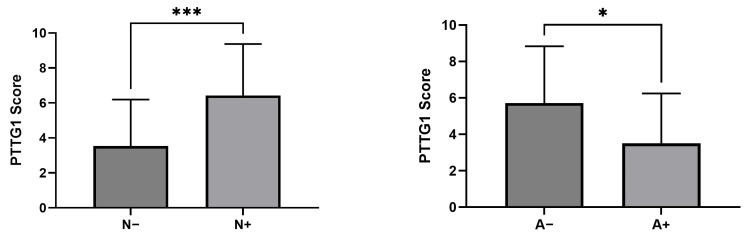
The relationships between the PTTG1 immunohistochemical score and clinicopathologic characteristics. The histograms show a significantly higher PTTG1 score in patients with lymph node metastases (N+) compared to those without nodal involvement (N−), and a significantly lower score in patients with adipose tissue invasion (A+) compared to those without invasion (A−). Statistical analysis was assessed using Student’s *t*-test for unpaired data: *** *p* < 0.01, * *p* < 0.05.

Figure 4 shows ROC curve for PTTG1 and lymph node metastasis.

**Figure 5:**
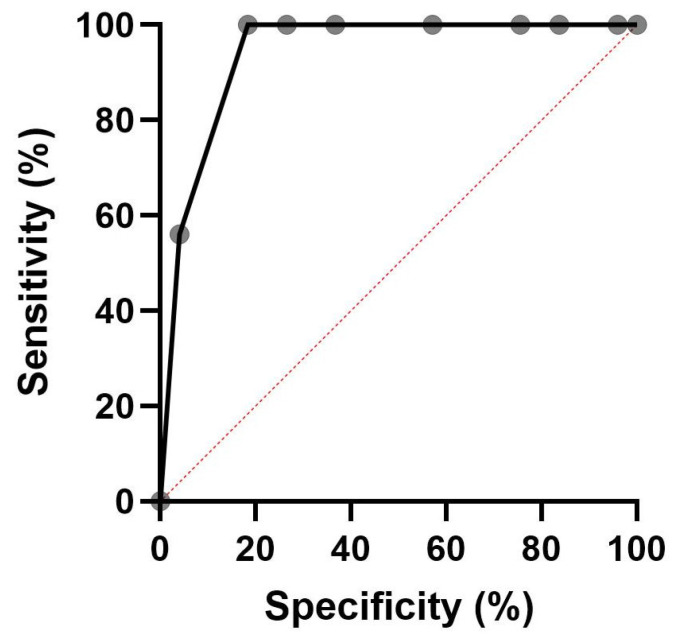
Receiver operating characteristic curve analysis of the PTTG1 immunohistochemical score for predicting lymph node metastases.

The ROC curve (Figure 5) illustrates the diagnostic performance of the PTTG1 immunohistochemical score in identifying patients with lymphadenopathy at diagnosis. The area under the curve (AUC) was 0.9392 (95% CI: 0.8898–0.9886; *p* < 0.001), indicating excellent discriminative ability. The optimal cut-off value, determined by Youden’s index, prioritized sensitivity over specificity to enhance the score’s potential clinical use as a prognostic marker.

To further evaluate the independent predictive value of the PTTG1 score for lymph node metastasis at diagnosis, we performed a binary logistic regression analysis including PTTG1 score (continuous variable), tumor size, and necrosis as covariates. These variables were selected based on both biological plausibility and prior univariate associations, while also considering sample size constraints. The overall model was statistically significant compared to the null model (Δχ^2^ = 21.29, df = 3, *p* < 0.001) and showed good explanatory power (Nagelkerke R^2^ = 0.473). Among the covariates, the PTTG1 score emerged as an independent predictor of lymphadenopathy (β = 0.502, *p* = 0.002), whereas tumor size did not reach statistical significance (*p* = 0.234). Necrosis was also significantly associated with lymph node metastasis (β = 1.925, *p* = 0.017). These results suggest that PTTG1 score correlates with N+ independently of tumor dimension and necrotic features. In the Supplementary Materials, Figure S1 illustrates the logistic regression.

## 4. Discussion

The present study represents a unique contribution, given that no work in the literature evaluates the clinical value of the proto-oncogene PTTG1 in testicular seminomas.

As already reported, the analysis of PTTG1 expression profiles in various TGCTs histotypes has shown large differences. Testicular teratomas and yolk sac tumors show no PTTG1 expression, while embryonal carcinomas exhibit high but chaotic expression. In contrast, testicular seminomas show a clear difference between the center and periphery, with increased intranuclear peripheral expression (involved in local invasion) and cytoplasmic expression in the central part of the neoplasm [14]. This finding was confirmed in the present work. Furthermore, the correlation between the expression of matrix metallo-proteinase 2 (MMP-2) in cell cultures was evident in seminoma cell cultures, while absent in embryonal carcinoma cell cultures [15]. The conclusion was that embryonal carcinoma, an aggressive tumor with a tendency for distant hematogenous metastases, does not depend on PTTG1 overexpression for its biological progression. Conversely, PTTG1 seems to play a central role in seminoma oncogenesis, promoting mechanisms of local invasiveness, followed by metalloproteinase expression [15] and dedifferentiation, and the induction of the epithelial–mesenchymal transition (EMT) [16]. In this regard, seminomas often present mutations in the KIT gene (encoding for c-Kit/CD117), a tyrosine kinase receptor expressed in germ cell lines and associated with the mitogen-activated protein kinase (MAPK) cascade, which in turn is closely linked to phosphorylation and activation of PTTG1 [24,25,26]. Moreover, among TGCTs, seminoma is the tumor most associated with lymph node metastases, while non-seminomatous tumors tend to metastasize via the hematogenous route [27]. In line with these findings, our work investigates the correlation between PTTG1 immunohistochemical profile and the presence of lymphadenopathy at diagnosis.

Immunohistochemistry using monoclonal antibodies for PTTG1 is already widely standardized and has been previously applied by our group [14]. The definition of the PTTG1 immunohistochemical score is based on highly relevant previously published studies [22]. The PTTG1 immunohistochemical score is directly correlated with lymph node involvement and inversely correlated with adipose tissue invasion. In light of the previously mentioned in vitro studies, the correlation between the PTTG1 immunohistochemical score and the presence of metastatic lymph nodes at diagnosis is supported by a solid pathophysiological rationale, whereas the inverse correlation with adipose tissue invasion appears to be a countertrend finding compared to the initial biological hypothesis. However, when comparing the ROC curves for the score’s predictive value with respect to these two parameters, it becomes evident that the score showed a good ability to discriminate patients with nodal involvement (good sensitivity, low specificity) reflecting a biologically aggressive phenotype (N+) at diagnosis while this role is lost for adipose tissue invasion (sensitivity of zero).

Given the hypothesized role of PTTG1 in local invasiveness and lymphatic dissemination, we tested whether the immunohistochemical PTTG1 score was independently associated with the presence of lymphadenopathy at diagnosis, after adjusting for potential confounders, each adjusting for a limited number of covariates to account for the sample size. Other than PTTG1 immunohistochemical score, a biological potential confounder as tumor size was considered, while necrosis was considered since it correlated with lymph node metastasis status according to preliminary analysis. The overall model was statistically significant compared to the null model and showed good explanatory power. Among the covariates, the PTTG1 score emerged as independently associated with lymphadenopathy, whereas tumor size did not reach statistical significance. Necrosis was also significantly associated with lymph node metastasis. These results suggest that PTTG1 score correlates with N+ independently of tumor dimension and necrotic features. Importantly, this finding should not be interpreted as the ability of PTTG1 to predict radiological lymphadenopathy per se, which is already detectable at diagnosis through standard imaging techniques. Rather, our results suggest that PTTG1 expression may serve as a tissue correlate of tumor aggressiveness, mirroring the biological processes underlying lymphatic spread.

The correlation between PTTG1 and lymph node metastasis has been explored in other tumors. Zhang E. et al. demonstrated that PTTG1 mRNA and protein levels in oral squamous cell carcinoma tissues correlated significantly with lymph node metastases and TNM stage. Additionally, in cell cultures, intranuclear PTTG1 correlated with increased MMP-2 expression and decreased E-cadherin expression (thus with EMT process), similar to what we previously demonstrated in testicular seminoma [28]. Analogous findings were reported in esophageal squamous cell carcinoma, where PTTG1 promotes EMT and correlates with lymph node metastasis [29,30], as well as in laryngeal carcinoma, where increased PTTG1 expression correlates with metalloproteinase expression and lymph node metastases [31]. Recently, an Italian group also correlated intranuclear PTTG1 levels with higher risk of lymph node metastases and aggressive phenotype in sporadic medullary thyroid carcinoma [32]. The hypothesis that PTTG1 increased nuclear expression leads to higher risk of lymph node metastasis is biologically plausible, since PTTG1 acts as a transcriptional stimulator of Vascular Endothelial Growth Factor (VEGF) [33,34], which is a key factor in lymphangiogenesis, and Fibroblast Growth Factor 1 (FGF1) [35]; in seminoma, PTTG1 has been clearly correlated with mechanisms favoring lymphatic invasion, such as metalloproteinase expression and EMT in cell cultures [16].

In testicular seminomas, the presence of lymphadenopathy correlates with a slightly worse prognosis and with a higher recurrence rate in seminoma patients [19,36]. Recurrence significantly impacts patient quality of life, both psychologically and physically, due to the need for further treatments [37]. One of the key questions in the management of stage I seminoma patients is the selection between adjuvant chemotherapy and active surveillance. Currently, this decision relies on risk factors, such as tumor size, angioinvasion, and/or rete testis invasion on histopathology [36,38,39,40]. In our cohort, no correlation was observed between PTTG1 score and tumor size, indeed, PTTG1 correlated with lymphadenopathy presence independently of tumor size. Rete testis invasion as a prognostic indicator has been questioned by recent meta-analyses [40]. Vascular invasion, although relevant in non-seminomatous tumors, is less important in seminoma; however, it is sometimes considered a recurrence risk factor [41].

The higher recurrence risk associated with active surveillance is due to undetectable micrometastases at diagnosis [42,43]. In this speculative context, PTTG1 could potentially reflect underlying biological features associated with occult lymphatic dissemination; however, this hypothesis cannot be addressed within the present study and requires dedicated investigation. Furthermore, in our work, the PTTG1 immunohistochemical score does not predict the recurrence rate. Further studies, with larger cohorts, are needed to test the hypothesis of PTTG1 score as a predictor of tumor recurrence and a feasible prognostic factor in testicular seminoma. Several studies have examined the role of PTTG1 as prognostic marker in other cancers with significant epidemiological impact. Read and colleagues found in 25 papillary thyroid carcinoma patients that high PTTG1 expression was associated with a 43% disease-free survival at 40 months compared to 87% in patients with low expression [44]. Associations between PTTG1 overexpression and worse prognosis have also been reported in papillary and clear cell renal carcinoma [45,46], bladder cancer [47], prostate adenocarcinoma [48,49,50], gliomas [51], hepatocellular carcinoma [52], adrenal tumors [53], gastric cancer [54], laryngeal carcinoma [31], and multiple myeloma [55]. All these studies assessed PTTG1 expression using molecular biology techniques (Western blot or mRNA quantification) or immunohistochemistry. Fina65

lly, recently, promising studies have explored serum PTTG1 measurement as an easy-to-use clinical biomarker [31,56].

Overall, our findings should be interpreted within the context of a preliminary, hypothesis-generating study. The study benefits from the multicenter design, a well-defined histopathological characterization, standardized immunohistochemical protocols, and the use of robust statistical methods, including ROC analysis and logistic regression, to evaluate predictive performance. Moreover, the cohort was homogeneous, with strict inclusion and exclusion criteria, thus reducing potential confounding due to tumor heterogeneity. However, some limitations and restrictions must be considered. While the association between PTTG1 expression and lymph node involvement is biologically plausible and supported by previous experimental data, the retrospective design and the limited sample size prevent definitive conclusions regarding clinical applicability. Furthermore, the studied population, with a high percentage of lymph node involvement, is not truly representative of the epidemiological distribution of patients affected by testicular seminoma. The use of radiological criteria for the assessment of lymph node involvement, without histological confirmation, may have led to a potential overestimation of metastatic disease, even though the radiological definition of metastatic lymphadenopathies was based on solid literature. A formal assessment of inter- and intra-observer variability for immunohistochemistry was not performed and represents a limitation of the study. Additionally, while the results are biologically plausible and supported by in vitro data, the cross-sectional nature of the analysis prevents conclusions regarding causality. Future prospective, larger-scale studies are warranted to confirm its predictive role and to better define its integration into clinical practice.

## 5. Conclusions

Our findings suggest that the PTTG1 immunohistochemical score may reflect biological features associated with lymphatic dissemination in testicular seminoma. While the score showed good sensitivity in identifying patients with nodal involvement, its clinical applicability remains to be established. Larger, prospective studies are required to validate its potential role as a biomarker in risk stratification.

## Figures and Tables

**Table 1:** Baseline demographic, clinical, and pathological characteristics of the study population. This table summarizes the baseline characteristics of the entire study cohort, including age, body mass index (BMI), tumor size, and PTTG1 immunohistochemical score. Quantitative variables are reported as mean ± standard deviation.

	Mean ± SD
**Age** (years)	40.6 ± 9.7
**BMI** (Kg/m^2^)	26.3 ± 3.4
**Tumor size** (cm)	4.3 ± 2.5
**PTTG1 Immunohistochemical score**	4.8 ± 3.1

**Table 2:** Tumor staging, histopathological features, treatment modalities, and recurrence rates in the study population.

**pTNM**	
pT1	7 (14%)
pT2	40 (78%)
pT3	4 (8%)
**Metastatic Lymphadenopathy**	
Yes	22 (43%)
No	29 (57%)
**Stage**	
I	29 (57%)
IIa	13 (25%)
IIb	4 (8%)
IIc	5 (10%)
**Rete testis invasion**	
Yes	37 (72%)
No	14 (28%)
**Vascular invasion**	
Yes	31 (61%)
No	20 (39%)
**Necrosis**	
Yes	21 (41%)
No	30 (59%)
**Adipose tissue and epididymal invasion**	
Yes	21 (41%)
No	30 (59%)
**Spermatic cord + resection margins**	
Positive	1 (2%)
Negative	50 (98%)
**Recurrence**	
Yes	7 (14%)
No	37 (72%)
Not Assessed	7 (14%)
**Therapies**	
Active Surveillance	11 (22%)
Carboplatin single shot	16 (31%)
PEB	12 (24%)
Radiation therapy	5 (10%)
Surgery	1 (2%)
Not Assessed	10 (19%)

## Data Availability

Data supporting the findings of this study are available from the corresponding author upon reasonable request.

## Supplementary Materials

The following supporting information can be downloaded at: https://www.mdpi.com/article/10.3390/cancers18071163/s1, Supplementary Table S1. Localization of the metastatic lymph nodes. Supplementary Figure S1. Logistic regression for metastatic lymph nodes and PTTG1 Score.

## Abbreviations

The following abbreviations are used in this manuscript:

TGCTs	testicular germ cell tumors
AFP	Alpha-fetoprotein
β-hCG	Beta-human chorionic gonadotropin
LDH	lactate dehydrogenase
PTTG1	Pituitary-Tumor-Transforming Gene 1
EMT	epithelial–mesenchymal transition
PLAP	placental alkaline phosphatase
DAB	diaminobenzidine
SD	standard deviation
MMP-2	Metallo-proteinase 2
MAPK	mitogen-activated protein kinase
VEGF	Vascular Endothelial Growth Factor
FGF1	Fibroblast Growth Factor 1
