# Supplementary material for: Intranuclear Peripheral Overexpression of Pituitary-Tumor-Transforming Gene 1: Immunohistochemical Biomarker of Lymph Node Involvement in Testicular Seminoma
Source: Cancers (Basel). 2026 Apr 4;18(7):1163. doi: 10.3390/cancers18071163 (PMC13072211; doi:10.3390/cancers18071163)
Supplement: Supplementary file 1 [file cancers-18-01163-s001.zip › cancers-4168148-supplementary.pdf]

Table S1. Localization of the metastatic lymph nodes. The table shows the localization of metastatic lymphoadenopathies, according to CT or MRI scan. N: Nodes (in TNM); NMN: Necrotic Matted Nodes; Nec: Necrosis; na: not assessed.

**Patient**	**Localization**	**Involved Sites (n)**	**Size (mm)**	**N**
**1**	Paraaortics	1	>10	cN1
**2**	Intercavoaortics	1	63	cN3
**3**	Paracavals<br>Intercavoaortics	2	16	cN1
**4**	Left Paraaortic	1	>10	cN1
**5**	Left Paraaortic	1	53	cN3
**6**	Left Paraortics<br>Intercavoaortics<br>Right External Iliac<br>Bilateral Inguinals (NMN)	4	44<br><10<br>na<br>na	cN2
**7**	Left Paraaortic	1	15	cN1
**8**	Right Paraaortic	1	>10	cN1
**9**	Left Crurals (NMN)	1	>10	cN1
**10**	Intercavoaortics	1	65	cN3
**11**	Intercavoaortics	1	70	cN3
**12**	Bilateral Paraaortics<br>Bilateral Intercavoaortics	2	14	cN1
**13**	Right inguinofemoral (Nec)	1	10	cN1
**14**	Left Obturators (NMN)	2	62	cN3
**15**	Bilateral Paraaortic<br>Bilateral Intercavoaortics<br>Right Paracaval<br>Bilateral External Iliacs (NMN)	4	22<br>na<br>na<br>17	cN2
**16**	Left Paraaortics	1	15	cN1
**17**	Left Paraaortics	1	16	cN1
**18**	Ileocolics (NMN)<br>Paracavals<br>Mesenterics (NMN)<br>Celiac tripod (NMN)	4	25<br>na<br>na<br>na	cN2
**19**	Left Paraaortic	1	11	cN1
**20**	Left Paraaortic	1	>10	cN1
**21**	Intercavoaortics	1	10	cN1
**22**	Intercavoaortics	1	43	cN2

Model Summary - N+ ▼

Model	Deviance	AIC	BIC	df	ΔX^2	p	McFadden R^2	Nagelkerke R^2	Tjur R^2	Cox & Snell R^2
M_0	66.92	68.925	70.817	48			0.000		0.000	
M_1	45.64	53.639	61.207	45	21.286	< .001	0.318	0.473	0.367	0.352

Note. M_1 includes Score, Dimensions, Necrosis

Coefficients

					Wald Test		
Model		Estimate	Standard Error	z	Wald Statistic	df	p
M_0	(Intercept)	−0.288	0.289	−0.997	0.993	1	.319
M_1	(Intercept)	−4.554	1.572	−2.896	8.389	1	.004
	Score	0.502	0.165	3.040	9.239	1	.002
	Dimensions	0.209	0.176	1.190	1.415	1	.234
	Necrosis	1.925	0.808	2.382	5.672	1	.017

Note. N+ level '1' coded as class 1.

Figure S1. Logistic regression for metastatic lymph nodes and PTTG1 Score. Binary logistic regression analysis including PTTG1 score, tumor size, and necrosis as covariates. The overall model was statistically significant compared to the null model ($\Delta\chi^2 = 21.29$, df = 3, $p < 0.001$), and showed

good explanatory power (Nagelkerke $R^2 = 0.473$). Among the covariates, the PTTG1 score emerged as an independent predictor of lymphadenopathy ($\beta = 0.502$, $p = 0.002$), whereas tumor size did not reach statistical significance ($p = 0.234$). Necrosis was also significantly associated with lymph node metastasis ($\beta = 1.925$, $p = 0.017$). These results suggest that PTTG1 score correlates with N+ independently of tumor dimension and necrotic features.
